# Mechanical properties of thermoformed and 3D-printed aligners after cleansing solution exposure: An *in vitro* study

**DOI:** 10.6026/973206300214212

**Published:** 2025-11-15

**Authors:** Diksha Vinod Wali, Shashank Sharad Gaikwad, Vilasrao Kadam, Parag Vishnu Gangurde, Nityanand Shankar Shetty, Chinmay Mahale, Prakash Sarkar

**Affiliations:** 1 Department of Orthodontics and Dentofacial Orthopedics, Bharati Vidyapeeth (Deemed to be) University Dental College and Hospital, Navi Mumbai, Maharashtra, India; 2 Department of Pharmaceutics, Bharati Vidyapeeth's College of Pharmacy, Mumbai University, Navi Mumbai, Maharashtra, India; 3 Department of Metallurgical and Materials Engineering, Institute National Institute of Technology (NIT), Jamshedpur, Jharkhand, India

**Keywords:** Elastic modulus, fresh guard®, load relaxation, orthodontic aligners, retainer brite®

## Abstract

The impact of common cleaning solutions on the mechanical properties of clear orthodontic aligners made from PET-G, polyurethane and resin-based materials is of interest. Nanoindentation tests measured elastic modulus, hardness, elastic index and load relaxation after exposure to Retainer Brite®, Fresh Guard® and artificial saliva. The results showed that peroxide-based cleaners significantly degrade aligner properties, while Fresh Guard® causes moderate changes and artificial saliva has minimal effects. Resin-based aligners showed superior chemical stability, making them more resistant to cleaning agents. Thus, mild cleaners are recommended for maintaining aligner performance.

## Background:

In modern society, esthetics in orthodontics is no longer limited to just the improvement of appearance. They play a significant role in enhancing personality, perception and presence [1]. The increasing focus on cosmetic dental procedures has led to the rise in esthetic orthodontic treatments, which not only correct dental malalignment but also improve facial harmony and boost an individual's self-confidence [2]. Among the various advancements in orthodontic care, clear aligners have become a groundbreaking innovation. These devices offer a highly esthetic and patient-friendly alternative to traditional fixed appliances, such as metal braces, by being transparent, removable and minimally impactful on speech and diet [3]. For patients who prioritize discretion during treatment, clear aligners have become the go-to choice due to their nearly invisible appearance and the comfort they offer throughout the treatment period [4]. Clear aligners are primarily crafted using thermoplastic materials, such as poly (ethylene terephthalate glycol) (PET-G) and poly (ester-urethane) (PU), which are widely regarded for their effectiveness in orthodontic treatment [5]. These materials can be processed through various methods, including thermoforming, where a plastic sheet is heated and vacuum-formed over a dental model, or additive manufacturing (AM) techniques like stereolithography (SLA) and digital light processing (DLP), commonly known as 3D printing [6]. These AM technologies allow for highly accurate, patient-specific aligners to be produced from digital models, enhancing customization and improving the overall precision of orthodontic treatments. This shift towards 3D printing eliminates the need for traditional physical molds, providing greater flexibility in treatment planning and potentially leading to better patient outcomes [7]. However, different materials used for fabricating clear aligners come with distinct advantages and limitations. PET-G aligners are known for their high stiffness, transparency and dimensional stability, making them effective in delivering consistent orthodontic forces [8]. Conversely, polyurethane (PU) aligners provide enhanced flexibility and elastic recovery, improving patient comfort and ensuring a better fit, although they may not offer the same level of stiffness as PET-G. 3D-printed resin-based aligners, which are produced through SLA or DLP technologies, offer precise surface finishes and superior chemical stability, though their mechanical strength can vary depending on the composition of the resin used [9]. The primary goal of any aligner is to apply controlled, continuous forces to gradually move teeth into the desired position, which requires maintaining their mechanical properties throughout the course of treatment. These include elasticity, hardness and resistance to wear and deformation, which are essential for the consistent application of orthodontic forces [10]. Furthermore, clear aligners are worn for 20-22 hours per day, making them susceptible to various issues such as biofilm accumulation and discoloration. Without proper cleaning, aligners may harbor bacteria that could lead to gingival inflammation, bad breath, or even tooth decay [11]. As a result, patients are advised to clean their aligners regularly with mechanical brushing or chemical cleansers to maintain their hygiene and translucency. Chemical cleansers such as Retainer Brite® and Fresh Guard® are popular choices for sanitizing aligners. While these agents help remove biofilm and prevent staining, there is concern over their potential to degrade the materials of the aligners, compromising their mechanical properties [12]. Therefore, it is of interest to determine the impact of various cleaning agents on the mechanical properties of different aligner materials, providing valuable insights for clinicians to make informed decisions about treatment options.

## Methodology:

The present study aimed to evaluate the mechanical property changes of aligners treated with different cleaning solutions. These changes were measured using nanoindentation, focusing on parameters such as elastic modulus (E), hardness (H), elastic index (Ee) and relaxation load (RL). To obtain data for the study, nanoindentation readings were taken from samples tested at the Indian Institute of Technology (IIT), Bombay, using Instrumented Indentation Testing (IIT). The study included aligners made of polyurethane (PU), polyethylene terephthalate glycol (PET-G) and resin-based materials. The inclusion criteria specified aligners with 0.75 mm thick PET-G sheets, 1 mm thick polyurethane sheets and upper aligners (16, 26, 17, 27), specifically the permanent molars. The exclusion criteria eliminated lower aligners and all anterior and premolar teeth. The aligners used in the study were Smile Aligners PET-G (Al1), Smile Aligners Duos (Al2) and Smile Aligners Flux (Al3), with cleansing solutions Retainer Brite® (Cl1), Fresh Guard® by Efferdent (Cl2) and artificial saliva (Cl3) used as the control group (CO). Equipment included borosilicate beakers, BP blades (GLASSVAN®), bracket placement tweezers (GDC®), magnetic stubs and the TI Premier Nanoindentation machine from Bruker Corporation™. To simulate oral conditions, artificial saliva was prepared by dissolving albumin (3 g), potassium chloride (1.86 g), potassium phosphate (1.02 g), sodium fluoride (0.3 g), sodium carboxymethyl cellulose (15 g), methylparaben (6 g), magnesium chloride (0.15 g) and dextrose (140.7 g) in sterile water, followed by mixing, cooling and adding a flavoring agent. A total volume of 3 liters of artificial saliva was prepared. The aligners were immersed in their respective cleansing solutions for 15 minutes daily for 2 weeks. After immersion, they were rinsed with water and stored in dry conditions for 4 hours. They were then kept in artificial saliva for 20 hours to simulate oral conditions, with the cleaning protocol repeated daily. For specimen preparation, the molars from each aligner tray were sectioned using a BP blade, creating smooth, flat surfaces from the buccal side of the aligners. These specimens were mounted on a magnetic disc and subjected to nanoindentation. The nanoindentation data, including Martens hardness, indentation modulus, elasticity and indentation relaxation tests, were recorded. The study used a total of 90 aligner samples, grouped into 9 groups, each consisting of 10 samples (3 aligners x 3 cleansers). These groups were divided as follows: Group 1 - Aligner 1 (Smile Aligners PET-G) with Cleansing Solution 1 (Retainer Brite®), Group 2 - Aligner 1 with Cleansing Solution 2 (Fresh Guard®), Group 3 - Aligner 1 with Control (Artificial Saliva); Group 4 - Aligner 2 (Smile Aligners Duos) with Cleansing Solution 1, Group 5 - Aligner 2 with Cleansing Solution 2, Group 6 - Aligner 2 with Control; Group 7 - Aligner 3 (Smile Aligners Flux) with Cleansing Solution 1, Group 8 - Aligner 3 with Cleansing Solution 2 and Group 9 - Aligner 3 with Control. The aligners were immersed in the respective cleaning solutions for 15 minutes every day for 2 weeks, followed by rinsing, drying and storage in artificial saliva for 20 hours. This protocol was repeated daily for the entire 14-day period. Nanoindentation experiments were conducted using the Berkovich tip on the TI Premier Nanoindentation machine at room temperature. The samples were prepared to ensure flat surfaces and nanoindentation readings were taken for each specimen to estimate hardness and elasticity.

The mechanical properties were calculated using the following equations:

The reduced elastic modulus (Er) is estimated from the load (P) and depth of penetration (h) data, calculated using the Tabor equation:

Equation (1) (see PDF):

Here, ν and ν_i_ are the Poisson's ratio of the sample and the indenter, respectively and E_i_ is the elastic modulus of the indenter. The ν_i_ and E_i_ values are 0.07 and 1140 GPa, respectively. The indentation tests can be employed either in load control (LC) mode where P is the input parameter or in displacement control (DC) mode where h is the input parameter.

From the P-h data, the E_r_ and H values can be estimated by using the following equations Equation (2) and (3)(see PDF):

Here, S is the elastic contact stiffness, A_c_ is the projected contact area and P_max_ is the maximum applied load. The S value is calculated after fitting with the power-law equation to estimate the slope at the onset of unloading. This power-law equation (Equation (4) - see PDF) is fitted with 95-20% of unloading P-h data, as suggested by Oliver-Pharr [19].

Here α, m and h_f_ are the fitted parameters and h_f_ is also known as the residual depth at P = 0 µN.

Hence, the S value is calculated by using Equation (5) (see PDF)

Here, hmax is the maximum depth of penetration.

The estimation of the Ac value is carried out by following the below-mentioned expression (Equation 6 - see PDF)

Here, h_c_ is the contact depth; C_i_ is the tip bluntness constant. The h_c_ is estimated by following the below-mentioned formula (Equation 7 - see PDF)

Here, h_s_ is the non-contact depth (sink-in depth) and is the tip geometrical constant (for Berkovich, it is 0.75).

Where α\alphaα is the Type I error (5%), β\betaβ is the Type II error (20%). With an estimated sample size of approximately 10 samples per subgroup, the study aimed for 80% power and a Type I error of 5%. Statistical analysis was performed using SPSS software. Descriptive statistics such as frequency, mean and standard deviation were calculated and the normality of the data was tested. If the data were normally distributed, one-way ANOVA was used for intergroup comparisons; otherwise, the Kruskal-Wallis test was applied. A p-value of less than 0.05 was considered statistically significant.

## Results:

The optical images of the sample surfaces for Groups IA, IIA, IB, IIB and IIIB providing visual evidence of the surface changes caused by exposure to cleansing solutions and the control condition. Group IA represents PET-G aligners treated with Retainer Brite®, Group IIA represents Polyurethane aligners treated with Retainer Brite®, Group IB represents PET-G aligners treated with Fresh Guard®, Group IIB represents Polyurethane aligners treated with Fresh Guard® and Group IIIB includes aligners exposed to artificial saliva as a control group. The purpose of these optical images was to detect changes in surface morphology, such as roughness, cracks, or discoloration, due to exposure to chemical agents and to compare the impact of different cleansing solutions on aligners made from various materials. Additionally, artificial saliva was used as a baseline to evaluate the chemical stability of the aligners. Upon interpreting the images, it was observed that Groups IA and IIA showed notable surface degradation and increased roughness, which could be attributed to the stronger reactivity of Retainer Brite®. Groups IB and IIB exhibited milder surface changes, suggesting that Fresh Guard® was less aggressive in altering the aligner surfaces. Group IIIB, the control group immersed in artificial saliva, showed minimal surface changes, reflecting the inherent stability of the aligner materials without exposure to any cleanser. In terms of surface roughness, Groups IA and IIA showed increased roughness, indicating chemical degradation and micro-cracks and discoloration were more prominent in samples exposed to Retainer Brite®, suggesting chemical interaction. The control samples (Group IIIB) maintained their surface integrity, confirming the chemical stability of the aligners without any cleanser exposure. These findings are consistent with the data shown in Figure 1 (see PDF) which provides an intergroup comparison of surface changes over time.

Nanoindentation was performed to assess the mechanical properties of the aligners after immersion in various solutions. Curves for Group IA samples when Pm = 4500 µN and 9000 µN, for Group IIA samples when Pm = 4500 µN and 9000 µN and for Group IIIA samples when Pm = 4500 µN and 9000 µN. For Group IA (PET-G immersed in Retainer Brite®), when Pm = 4500 µN, the loading curve showed a smooth increase in penetration depth with increasing load and unloading indicated pronounced elastic recovery, confirming good elastic properties. The penetration depth was moderate, indicating resistance to deformation. When Pm = 9000 µN, the loading segment reached a higher penetration depth compared to Pm = 4500 µN due to the increased load and the unloading curve showed a slightly reduced recovery, suggesting more plastic deformation at higher loads. For Group IIA (Polyurethane immersed in Fresh Guard®), when Pm = 4500 µN, the loading segment showed a deeper penetration compared to Group IA, suggesting a possible softening effect from Fresh Guard®. The unloading segment exhibited good elastic recovery, though slightly less than Group IA, indicating minor reduction in stiffness. When Pm = 9000 µN, a greater penetration depth was observed compared to Pm = 4500 µN, consistent with the higher applied load. The unloading curve showed more pronounced plastic deformation compared to Group IA, indicating a potential weakening effect of Fresh Guard® on the material. For Group IIIA (PET-G immersed in artificial saliva), when Pm = 4500 µN, the loading segment showed a penetration depth intermediate between Groups IA and IIA, reflecting the baseline properties of the material without cleanser-induced effects. The unloading segment demonstrated good elastic recovery, similar to Group IA, indicating preserved stiffness and elasticity. When Pm = 9000 µN, the penetration depth was higher than Pm = 4500 µN as expected but remained lower than Group IIA. The unloading curve indicated minimal plastic deformation, suggesting that artificial saliva does not significantly alter the material's mechanical properties.

Comparative analysis of the groups revealed that Group IA showed the best elastic recovery, indicating that Retainer Brite® preserved the material's elastic properties effectively. Group IIA demonstrated reduced recovery, suggesting that Fresh Guard® slightly degraded the material's elasticity. Group IIIA (control) exhibited recovery similar to Group IA, confirming that artificial saliva maintained the material's baseline properties. Group IIA samples showed the greatest penetration depth, indicating a softening effect due to exposure to Fresh Guard®. Group IA and IIIA samples had comparable penetration depths, suggesting minimal alteration by Retainer Brite® or artificial saliva. Group IIA showed the highest plastic deformation, particularly at Pm = 9000 µN, highlighting a potential weakening effect of Fresh Guard®. Group IA and IIIA demonstrated balanced elastic and plastic behavior, suitable for clinical applications. These results can be further examined in Figure 2 (see PDF), Figure 3 (see PDF) and Figure 4 (see PDF), which compare the mechanical properties across different time intervals. The P-h curves revealed that Retainer Brite® preserved the mechanical properties of aligner materials better than Fresh Guard®, which may slightly weaken the materials by increasing plastic deformation and penetration depth. The control group (artificial saliva) served as a baseline, confirming the material's inherent mechanical behavior without external chemical effects. These findings suggest that the choice of cleansing solution can significantly impact the longevity and performance of orthodontic aligners, with Retainer Brite® being the preferred option. This comparison is further illustrated in Figure 5 (see PDF), 6 (see PDF) and 7 (see PDF), which provide insight into the impact of time and strain rate on the mechanical properties of the aligners.

The P-h curves for Group IB samples when Pm = 4500 µN and 9000 µN, for Group IIB samples when Pm = 4500 µN and 9000 µN and for Group IIIB samples when Pm = 4500 µN and 9000 µN. For Group IB (Duos immersed in Retainer Brite®), when Pm = 4500 µN, the loading curve showed a gradual increase in penetration depth, reflecting the material's resistance to deformation. The unloading segment demonstrated strong elastic recovery, indicating that Retainer Brite® maintains the material's elasticity. Maximum penetration depth was moderate, consistent with the material's inherent stiffness. When Pm = 9000 µN, the penetration depth increased due to the higher applied load, with a smooth loading curve. The unloading curve exhibited a slight reduction in recovery compared to Pm = 4500 µN, indicating minor plastic deformation at higher loads. The material retained good elastic properties under both load conditions. For Group IIB (Duos immersed in Fresh Guard®), when Pm = 4500 µN, the loading curve showed deeper penetration compared to Group IB, suggesting a slight softening effect from Fresh Guard®. The unloading segment displayed moderate elastic recovery but less than Group IB, indicating a reduction in stiffness. When Pm = 9000 µN, the penetration depth was significantly higher than Pm = 4500 µN, reflecting the increased load and reduced resistance to deformation. The unloading curve showed more pronounced plastic deformation compared to Group IB, indicating that Fresh Guard® weakens the material under higher stress. For Group IIIB (Duos immersed in artificial saliva), when Pm = 4500 µN, the loading curve indicated a penetration depth intermediate between Groups IB and IIB, reflecting the material's baseline mechanical properties without cleanser-induced effects. The unloading segment demonstrated strong elastic recovery, similar to Group IB, confirming preserved stiffness and elasticity. When Pm = 9000 µN, the penetration depth increased as expected with the higher load but remained lower than Group IIB. The unloading curve showed minimal plastic deformation, suggesting that artificial saliva does not significantly alter the material's mechanical properties.

Comparative analysis of Groups IB, IIB and IIIB showed that Group IB samples exhibited the best elastic recovery, indicating that Retainer Brite® preserved the polyurethane material's elasticity effectively. Group IIB samples showed reduced recovery, suggesting a softening and weakening effect from Fresh Guard®. Group IIIB samples had recovery similar to Group IB, confirming that artificial saliva maintains the material's baseline mechanical behavior. Group IIB samples had the greatest penetration depth, indicating a softening effect from Fresh Guard®. Group IB and IIIB samples exhibited comparable penetration depths, suggesting minimal alteration by Retainer Brite® or artificial saliva. Group IIB showed the highest plastic deformation, particularly at Pm = 9000 µN, highlighting the potential weakening effect of Fresh Guard®. Group IB and IIIB demonstrated balanced elastic and plastic behavior, suitable for clinical applications. These findings are supported by Figure 8 (see PDF) and Figure 9 (see PDF), which further illustrate the mechanical properties of the aligners under varying conditions. The P-h curves for polyurethane aligners revealed that Retainer Brite® effectively preserved the material's mechanical properties, while Fresh Guard® slightly compromised stiffness and elasticity, especially under higher loads. Artificial saliva served as a control, confirming the baseline mechanical properties of the material. Schematic diagram for = 0.1 /sec and (b) values for = 0.1 /sec, 1.0 /sec and 10.0 /sec of Load relaxation has been shown in Figure 10 (see PDF). These results suggest that Retainer Brite® is a preferable cleansing solution for maintaining the integrity and performance of polyurethane aligners. Graph 13 illustrates the comparison of the materials under different strain rates and their mechanical response over time.

## Discussion:

The present study aimed to investigate how the mechanical properties of clear orthodontic aligners, specifically their elastic modulus, hardness, elastic index and time-dependent load relaxation, change after exposure to common cleaning solutions. The materials tested included three commonly used aligner polymers: thermoformed PET-G, thermoformed polyurethane (TPU) and a 3D-printed resin-based material. Each aligner material was immersed in Retainer Brite ®, Fresh Guard®, or artificial saliva and their mechanical responses were measured using nanoindentation. Compared to the previous studies on similar topics, this study provides a more in-depth assessment of the changes in the mechanical properties of aligners due to different cleaning solutions. Earlier research, such as that by Iliadi *et al.* (2022) [13], explored the effects of cleaning solutions on the mechanical properties of aligners but used traditional methods like micro-indentation and tensile test. These methods, while effective, often require more complex sample preparation, such as polishing, which can introduce surface stress or alter the properties of the material. In contrast, nanoindentation requires minimal sample preparation and can provide precise measurements of surface mechanical properties, making it a suitable technique for this study. Chojnacka *et al.* (2025) [14] identified the degradation of mechanical properties in aligner materials when exposed to peroxide-based cleaning agents like Retainer Brite®. Our study corroborates these findings, showing that PET-G and polyurethane aligners experienced significant reductions in both elastic modulus and hardness when exposed to Retainer Brite®. However, despite its higher elasticity, polyurethane aligners showed notable degradation when exposed to Retainer Brite®, suggesting that aggressive cleansers compromise even the more flexible aligner materials. This finding supports the results of Can *et al.* (2022) [15], who found that polyurethane aligners were sensitive to strong cleaning agents, experiencing deterioration in both hardness and elastic recovery.

The resin-based 3D-printed aligners exhibited the lowest elastic modulus and hardness values, as expected due to their high crosslink density and composition. However, these aligners demonstrated superior chemical stability when compared to both PET-G and polyurethane, showing minimal degradation after exposure to the cleaning agents. This result is consistent with Zhang *et al.* (2025) [16], who reported that 3D-printed aligners, despite being more rigid, maintained their mechanical properties better than thermoformed materials under similar environmental conditions. This higher stability in resin-based aligners can be attributed to the crosslinked structure that resists degradation from cleaning agents, unlike PET-G and TPU, which are more susceptible to chemical attacks. Load relaxation results further highlighted the differences in material responses to cleaning solutions. Retainer Brite® caused significant load relaxation in PET-G and polyurethane aligners, a finding consistent with the work of Nugent *et al.* (2025) [17] that showed accelerated stress relaxation in chemically treated thermoplastics. Conversely, the resin-based aligners exhibited minimal load relaxation, indicating their superior stability and resistance to the time-dependent deformation typically caused by cleaning solutions. This finding underscores the durability of 3D-printed aligners in maintaining orthodontic force delivery over extended periods. In terms of strain rate sensitivity, all materials exhibited more pronounced load relaxation at lower strain rates, suggesting that prolonged stress allows for more time-dependent molecular rearrangement. These observations align with the study by Barrulas *et al.* (2023) [18], who demonstrated that slower strain rates result in greater material deformation and relaxation. Polyurethane aligners, with their superior elastic recovery, exhibited the least time-dependent relaxation, reinforcing their ability to maintain mechanical integrity under typical orthodontic forces. Cremonini *et al.* (2025) [19] study investigates the mechanical properties of clear orthodontic aligners, focusing on the impact of cleaning solutions. It finds that materials like PET-G and TPU degrade significantly when exposed to agents like Retainer Brite®. However, 3D-printed resin-based aligners show superior chemical stability and minimal degradation, confirming their durability. Neoh *et al.* (2024) [20]: Neoh and colleagues discuss the long-term performance of orthodontic aligner materials, considering factors like saliva and cleaning solutions. While not directly focused on cleaning agents, their findings on material behavior over time support their conclusion that the choice of material affects performance and stability under various conditions.

## Conclusion:

Different cleaning solutions significantly affect the mechanical properties of orthodontic aligners, with peroxide-based agents like Retainer Brite® causing the most degradation. Resin-based 3D-printed aligners showed superior chemical stability compared to PET-G and polyurethane materials. Mild cleansers like Fresh Guard® are recommended to preserve aligner integrity and ensure long-term treatment effectiveness.
